# Supplementary material for: Impact of historical land use change on the brown bear habitat connectivity in the Polish Carpathians
Source: PeerJ. 2025 Nov 11;13:e20295. doi: 10.7717/peerj.20295 (PMC12617368; doi:10.7717/peerj.20295)
Supplement: Supplemental Information 4 [file peerj-13-20295-s004.docx]

**Supplementary material**

**Impact of historical land use change on the brown bear habitat connectivity in the Polish Carpathians**

Anna Szwagierczak^1^, Elżbieta Ziółkowska^1,2,3^, Joanna Wąs^4^, Michał Jakiel^1^, Dominik Kaim^1^

^1^Institute of Geography and Spatial Management, Faculty of Geography and Geology, Jagiellonian University, Gronostajowa 7, 30-387 Kraków, Poland

^2^Institute of Environmental Sciences, Faculty of Biology, Jagiellonian University, Gronostajowa 7, 30-387 Kraków, Poland

^3^Social-Ecological Systems Simulation Centre, Department of Agroecology – Agricultural biodiversity, Aarhus University, Aarhus, Denmark

^4^Institute of Geography and Spatial Organization Polish Academy of Sciences, Department of Geoenvironmental Research, św. Jana 22, 31-018, Kraków, Poland

Corresponding Author:

Anna Szwagierczak^1^

Gronostajowa 7, 30-387 Kraków, Poland

Email address: anna.szwagierczak@student.uj.edu.pl

Tab.S1. Comparison of estimated original coefficients for the most important variables representing three group of factors (forest-related, human impact-related and topography-related) used by other authors working in the northern part of the Carpathians. The original coefficients were transformed into absolute values and standardised into up to 4 categories (‘Standardized rank’ column), where the class limit values were the quartiles assessed by using Tukey method (1 – ≤ Q1, 2 – (Q1, Q2],
3 – (Q2, Q3], 4 – > Q3).

| **Factor** | | **Original coefficient value** | **Rank units** | **Scope** | **Spatial resolution, area, period** | **Reference** | **Standa-rized rank**  **(1-4)** |
| --- | --- | --- | --- | --- | --- | --- | --- |
| FOREST-related | Percentage of forest in surrounding area (325km^2^) | 1.383 | Scaled coefficient estimates | Model of an occurrence probability | 5x5km cells, Poland (mostly Carpathians), based on bear observations from 1985–2005 | Fernández et al., 2012 | 3 |
| HUMAN IMPACT-related | Human density in surrounding area (750km^2^) | -0.741 |  |  |  |  | 1 |
|  | Urban areas in surrounding area (750km^2^) | -1.003 |  |  |  |  | 2 |
| FOREST-related | Forest | 0.8613 | Average probabilities of bear occurrence | Model of  a habitat suitability | 30x30m cells, Slovakian Carpathians, based on bear presence within hunting grounds in 2008 | Koren et al., 2011 | 4 |
|  | Forest and buffer zone | 0.6752 |  |  |  |  | 3 |
|  | Non-forested land | 0.2866 |  |  |  |  | 2 |
|  | Non-forested land without buffer around forest | 0.0365 |  |  |  |  | 1 |
| FOREST-related | Percentage of forest within  a 0.25 km distance | 0.481 | Mean coefficient estimates | Model of  a movement suitability | 30x30m cells, Polish and Slovakian Carpathians, based on telemetry data in 2008–2009 and 2014–2015 | Ziółkowska et al., 2016 | 2 |
|  | Deciduous to total forest ratio within  a 2 km distance | -2.689 |  |  |  |  | 2 |
|  | Percentage of mixed forest within a 0.5 km distance | 0.384 |  |  |  |  | 1 |
|  | Percentage of forest edge within a 0.25 km distance | 0.433 |  |  |  |  | 1 |
| TOPOGRPAHY-related | Mean elevation range within a 0.25 km distance | 3.767 |  |  |  |  | 3 |
| HUMAN IMPACT-related | Density of roads within a 0.25 km distance | -5.911 |  |  |  |  | 4 |
|  | Density of settlements within  a 0.5 km distance | -5.711 |  |  |  |  | 3 |

Tab. S2 Summarized standardized ranks from the table S1 referred to the coefficients used in this work (weighted variant), where the costs were also standardized using the same method and ranks, as in the Tab. S1.

| Factors | Rank (higher value indicates importance in the model) | | | | | | | |
| --- | --- | --- | --- | --- | --- | --- | --- | --- |
|  | This work  (weighted variant) | | Fernández et al., 2012 | | Koren et al., 2011 | | Ziółkowska et al., 2016 | |
|  | cost range | rank | coefficients | rank | coefficients | rank | coefficients | rank |
| FOREST-related | 1-25 | 1-4 | 1.383 | 3 | 0.8613,  0.6752,  0.2866,  0.0365, | 1-4 | 0.481  -2.689  0.384  0.433 | 1-3 |
| HUMAN IMPACT-related | 0-20 | 1-3 | -0.741,  -1.003 | 1-2 |  | - | -5.911,  -5.711 | 3-4 |
| TOPOGRAPHY-related | 2-20 | 1-3 | - | - |  | - | 3.767 | 3 |


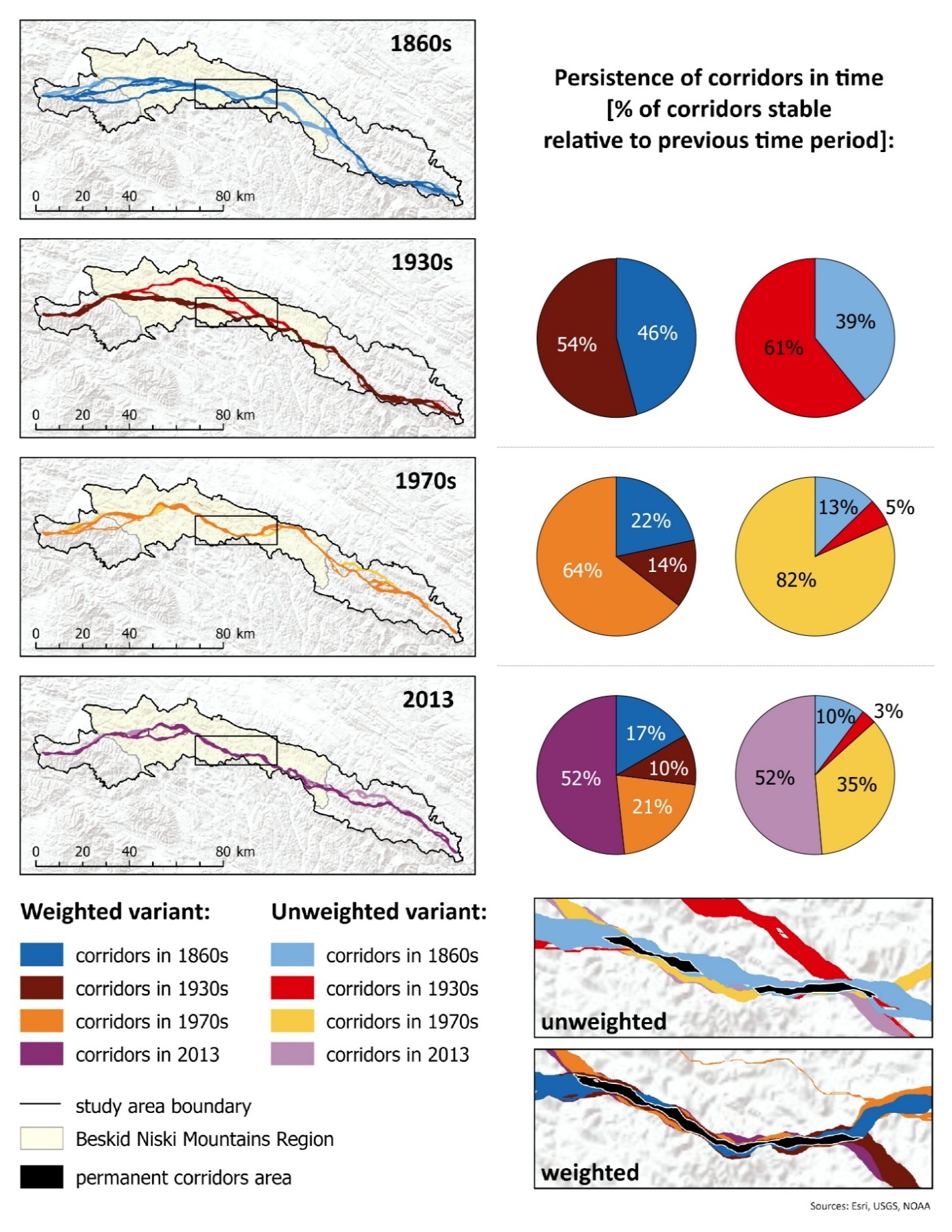


Fig.S1. Brown bear corridors connecting Beskid Sądecki in the west and Bieszczady in the east through the Beskid Niski Mts based on 10^th^ percentile. Colours represent different variants (unweighted vs. weighted) of cost surfaces and analysed time periods (1860s, 1930s, 1970s and 2013).
*Digital elevation model © ESRI, USGS, NOAA*


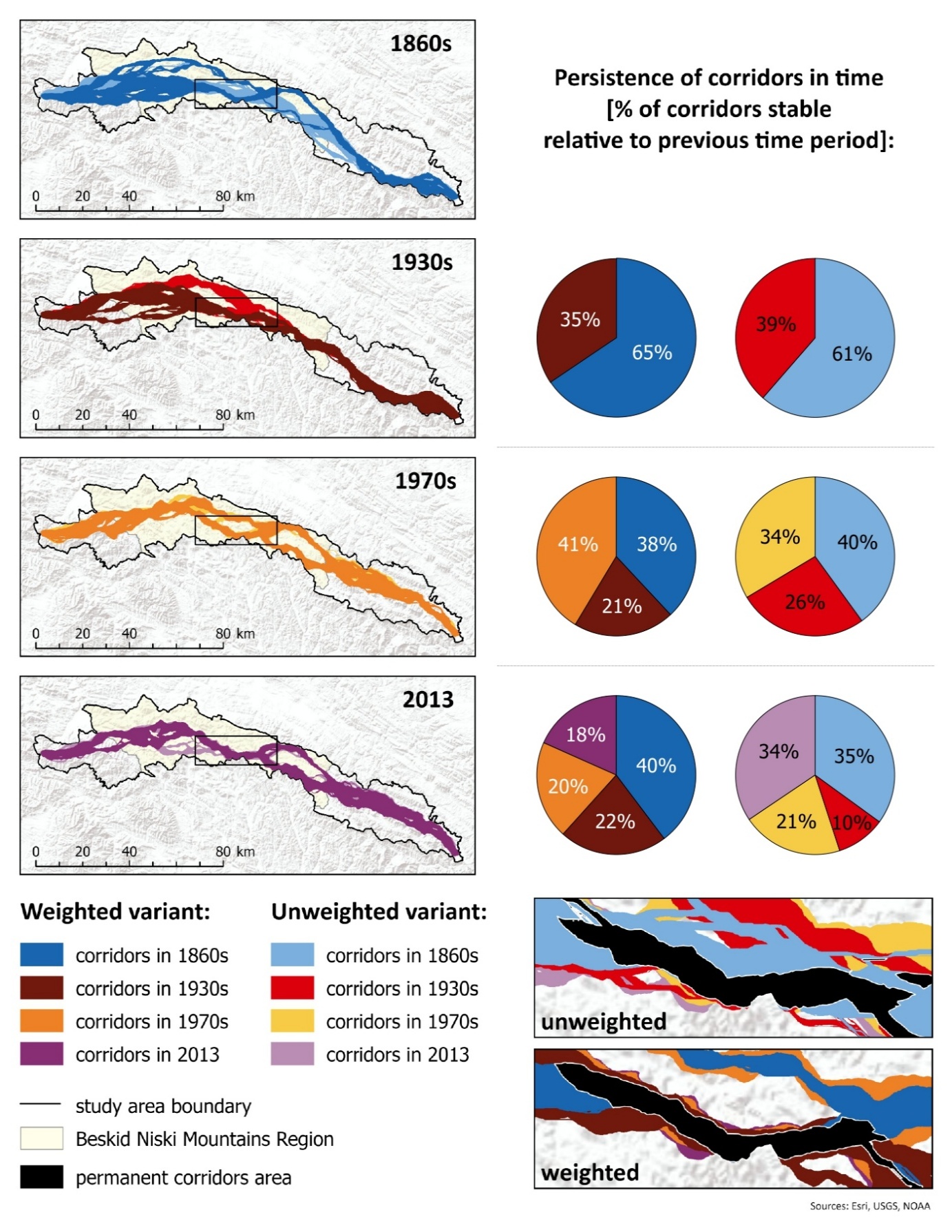


Fig.S2. Brown bear corridors connecting Beskid Sądecki in the west and Bieszczady in the east through the Beskid Niski Mts based on 30^th^ percentile. Colours represent different variants (unweighted vs. weighted) of cost surfaces and analysed time periods (1860s, 1930s, 1970s and 2013).
*Digital elevation model © ESRI, USGS, NOAA*


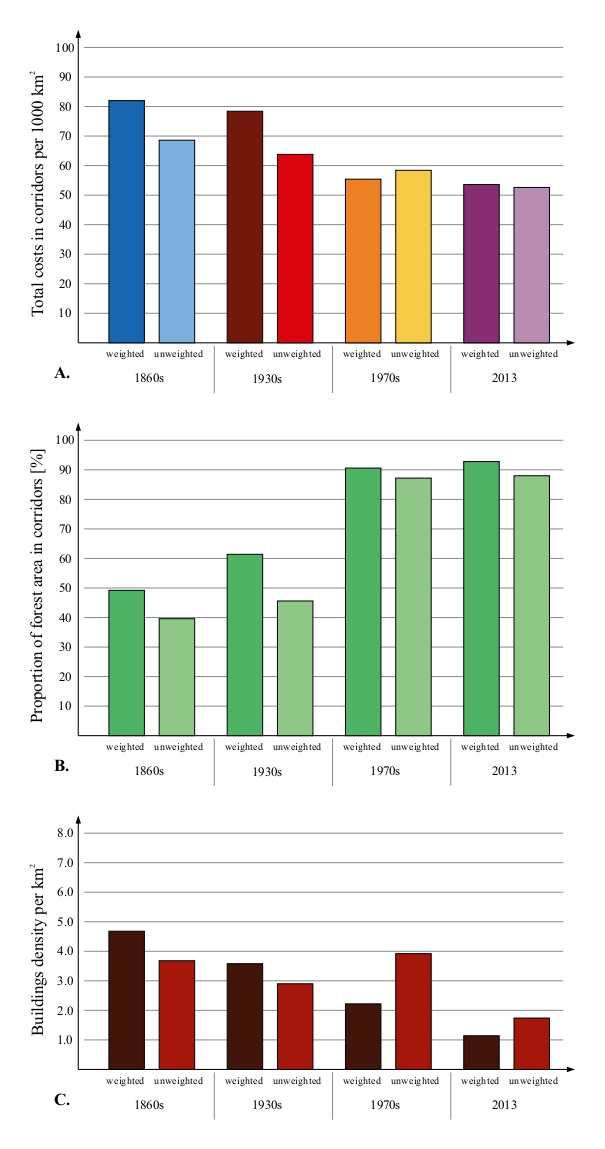


Fig.S3. Total costs in corridors (based on 10^th^ percentile) related to the area of corridors (per m^2^) (A), proportion of forest area in corridors (B) and buildings density per km^2^ (C).


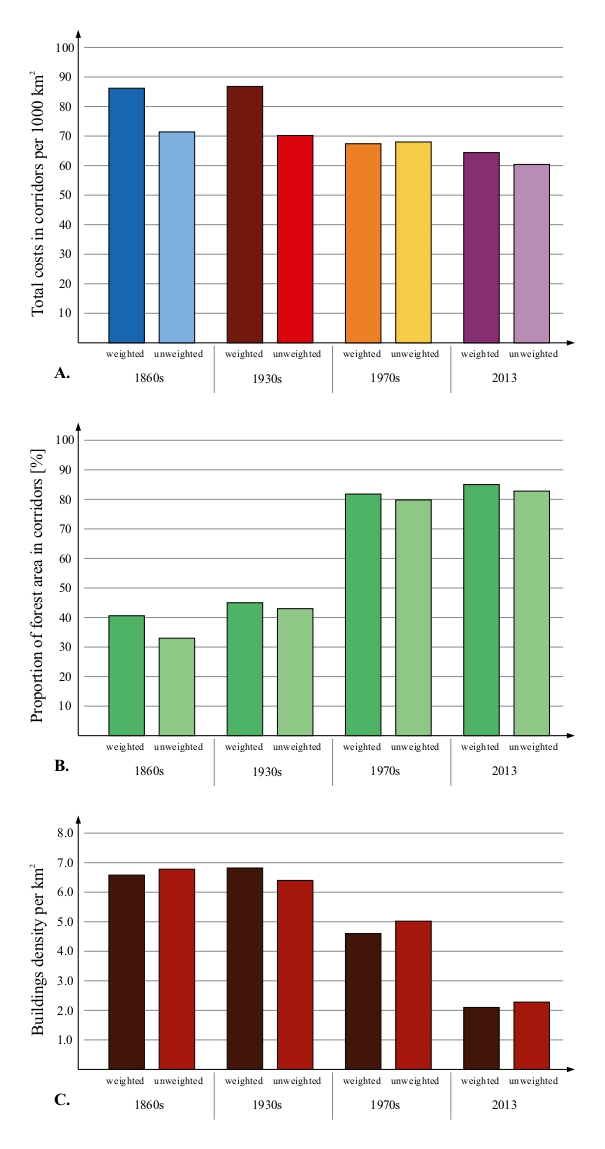


Fig.S4. Total costs in corridors (based on 30^th^ percentile) related to the area of corridors (per m^2^) (A), proportion of forest area in corridors (B) and buildings density per km^2^ (C).


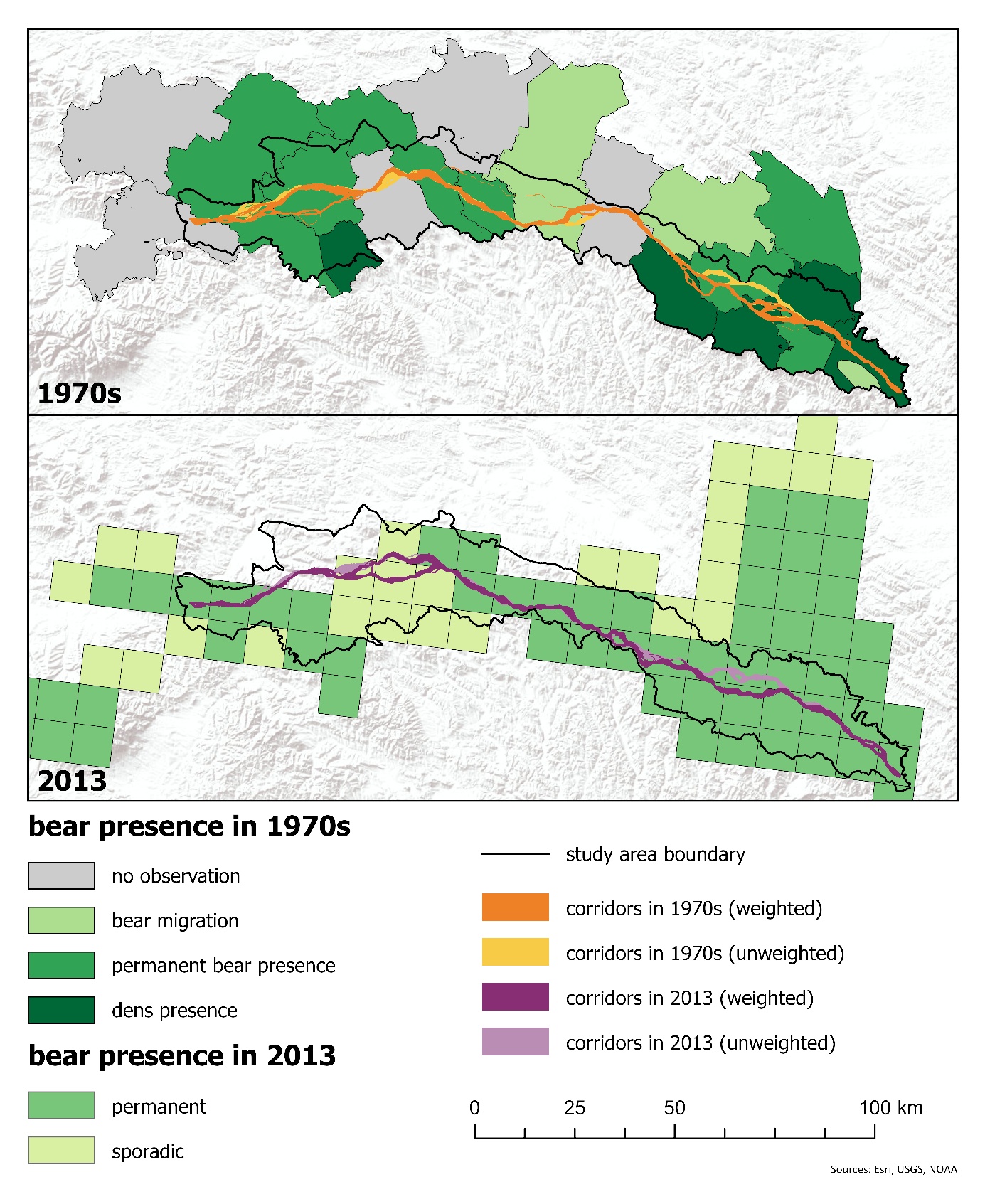


Fig.S5. Corridors based on 10^th^ percentile referred to the bear presence data for 1970s and 2010s. *Sources for bear occurrence data: 1970s: (Jakubiec & Buchalczyk, 1987), 2010s: (Chapron et al., 2014).* *Digital elevation model © ESRI, USGS, NOAA*


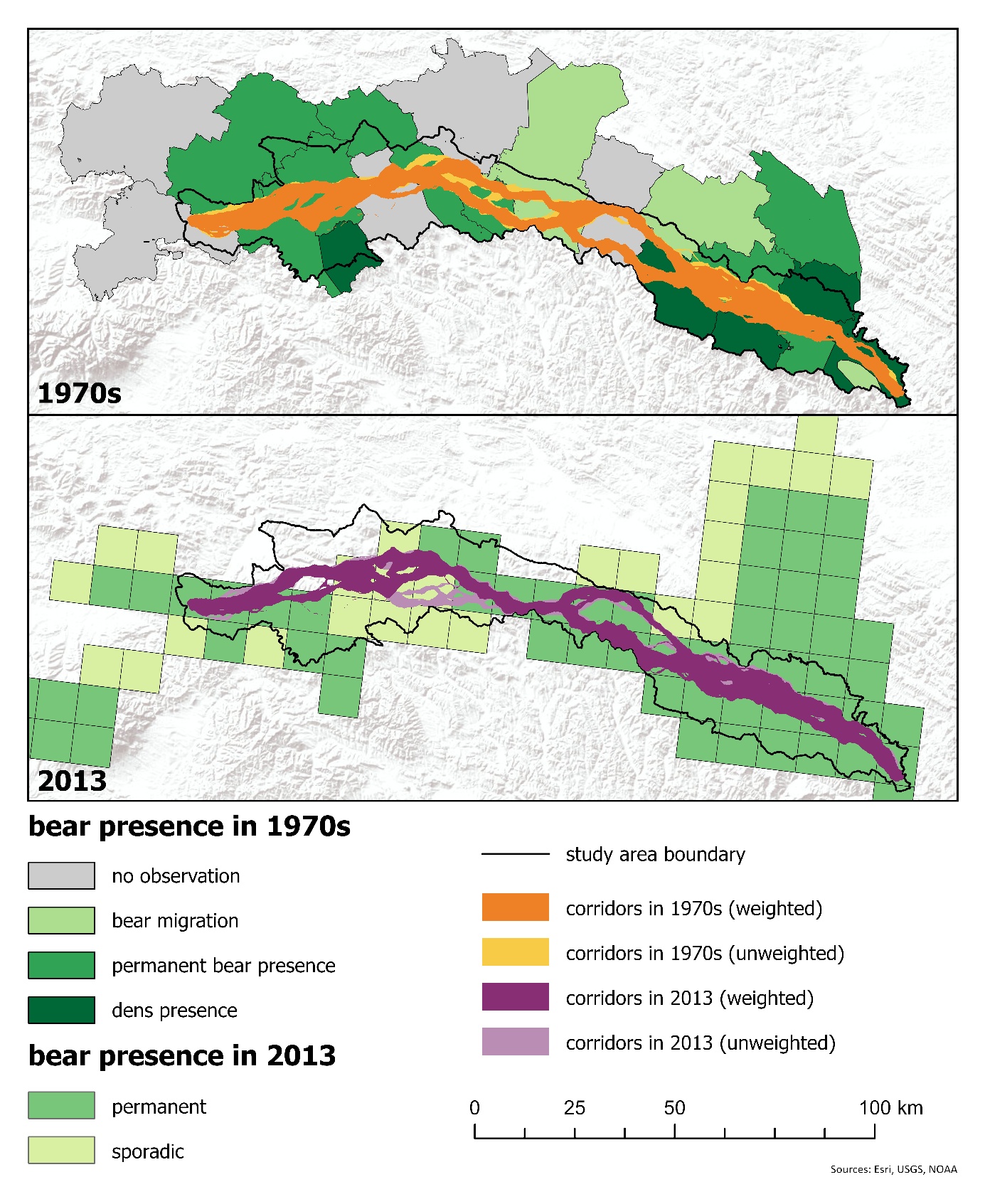


Fig.S6. Corridors based on 30^th^ percentile referred to the bear presence data for 1970s and 2010s. *Sources for bear occurrence data: 1970s: (Jakubiec & Buchalczyk, 1987), 2010s: (Chapron et al., 2014).* *Digital elevation model © ESRI, USGS, NOAA*
